# Humans rely more on algorithms than social influence as a task becomes more difficult

**DOI:** 10.1038/s41598-021-87480-9

**Published:** 2021-04-13

**Authors:** Eric Bogert, Aaron Schecter, Richard T. Watson

**Affiliations:** Management Information Systems Department, University of Georgia, Athens, GA 30602 USA

**Keywords:** Computer science, Information technology

## Abstract

Algorithms have begun to encroach on tasks traditionally reserved for human judgment and are increasingly capable of performing well in novel, difficult tasks. At the same time, social influence, through social media, online reviews, or personal networks, is one of the most potent forces affecting individual decision-making. In three preregistered online experiments, we found that people rely more on algorithmic advice relative to social influence as tasks become more difficult. All three experiments focused on an intellective task with a correct answer and found that subjects relied more on algorithmic advice as difficulty increased. This effect persisted even after controlling for the quality of the advice, the numeracy and accuracy of the subjects, and whether subjects were exposed to only one source of advice, or both sources. Subjects also tended to more strongly disregard inaccurate advice labeled as algorithmic compared to equally inaccurate advice labeled as coming from a crowd of peers.

## Introduction

Algorithms have mastered checkers^1^, chess^2,3^, poker^4^, and tasks with fewer boundaries such as information search^5^. This expertise has led humans to rely heavily on algorithms. For example, people rely so heavily on Google that they treat it as an external memory source, resulting in them being less able to remember searchable information^6^. As big data has flourished, people have become so comfortable with algorithms that drivers will sleep in their self-driving cars^7^, go on dates with algorithmically-recommended matches^8^, and allow algorithms to run their retirement accounts^9^. However, there are some tasks for which humans prefer to take advice from other humans, such as in medical advice^10^ or predicting how funny a joke will be^11^.

Humans often demonstrate greater reliance on advice from algorithms compared to non-algorithmic advice, exhibiting *algorithmic appreciation*^12^. Relying upon algorithms for analytical tasks is typically advantageous. Even simple algorithms, such as weighting all variables equally, can outperform human prediction^13^. In a meta-analysis of 136 studies, algorithms were 10% more accurate, on average, than non-algorithmic (human) judgment^14^. Consequently, for analytical tasks, we would expect a rational human to demonstrate algorithmic appreciation.

Of course, much of human behavior is not strictly rational^15^. People tend to discount or disregard advice, even when it is not logical to do so^16^. Often, the source of advice dictates how much it is discounted. When people discount advice from other people less than they discount advice from algorithms, particularly after observing an algorithm make a mistake, they demonstrate *algorithmic aversion*—the opposite of algorithmic appreciation. There is evidence for both algorithmic aversion^17^ and appreciation^12,18,19^, and it is task dependent^11^. Prior research has also shown that people rely on advice more heavily when tasks become more difficult^20^. However, this effect may not be uniform across sources of advice.

Given these empirical observations, we question whether task difficulty is an important explanatory variable in determining whether people demonstrate algorithmic appreciation or aversion. In our studies of reliance on algorithmic advice, we consider two critical factors: the source of advice and task difficulty. We conducted three preregistered experiments with N = 1500 participants to test the influence of algorithmic advice, compared to social influence, on human decision making. Broadly speaking, social influence encapsulates the myriad ways that humans change their behavior based on the actions of other people. Prior experiments show that when humans are exposed to social influence, the wisdom of the crowd can be reduced^21^, and that the structure of the social network dictates how social influence affects decision-making^22^. Based on subject responses across multiple tasks and under different manipulation conditions, we find that people rely more on algorithmic relative to social advice, measured using Weight on Advice (WOA)^23^. Further, we establish that advice acceptance varies as tasks increase in objective difficulty and as advice varies in quality.

## Results

In each experiment, subjects were asked how many people were in a photograph and provided advice that was purported to be from either “an algorithm trained on 5000 images” or “the average guess of 5000 other people.” There was no other introduction to the algorithm or a description of what types of people made the estimates. An equal number of subjects were in each group. We used a large group of peers as a reference group because large groups often makes guesses that are accurate, on average^21,24,25^, and people respond more strongly to advice from large numbers of people compared to advice from a single person^26^. We chose to design the experiment such that the only difference between the two sources of advice was the label, so that we could isolate the effect of advice source. This is a common method of judging reliance on algorithmic advice^11,12^.

We use the Judge Advisor System (JAS) in every experiment. The JAS is an experimental method in which subjects answer a question, are provided advice related to that question, and then asked to answer the question again^27–30^. In experiments using the JAS, a common dependent variable is Weight on Advice (WOA). WOA calculates the degree to which an individual changes their answer towards the advice, and thus is a useful measure for describing the extent of algorithmic appreciation or aversion.

All tests described below are two-tailed at the alpha 0.05 significance level and are *t* tests of coefficient values from a regression. Summary statistics of the data can be found in Table S1.

### Experiment 1: Advice as between subjects treatment

In the initial experiment, subjects were asked to determine how many people were in a picture and received advice that was labeled as either algorithmic or the average of human guesses, and they never received advice from the other source. All advice was the true answer, which was determined by the publisher of the dataset^31^.

### Task validation and randomization check

We first assessed whether subjects responded to more difficult problems by taking more time, being less confident, and being less accurate. When comparing within-person easy to hard questions, individuals are significantly more accurate (*t* = 2.745; *P* = 0.006; 95% confidence interval (CI) = 0.281 to 1.684), more confident ($t = 24.291$; *P* < 0.001; 95% CI = 0.536 to 0.630) and take less time ($t = 4.179$; *P* < 0.001; 95% CI = 0.041 to 0.113) for easy problems. In all three models we observed that subjects relied more on advice in difficult questions. Subjects placed more weight on advice for hard questions in our baseline model (*B* = 0.150; *P* < 0.001; 95% CI = 0.134 to 0.167), in the model including hypothesized interactions (*B* = 0.132; *P* < 0.001; 95% CI = 0.108 to 0.155), and in the model including all interactions and control variables (*B* = 0.081; *P* < 0.001; 95% CI = 0.057 to 0.105). Thus, we conclude that subjects perceived the relative difficulty of the questions as designed.

We compared the average initial accuracy, initial confidence, and initial time taken across treatments using a two-sample t-test. For individuals exposed to algorithmic advice, there was not a statistically significant difference in initial accuracy ($t = - 0.767$; *P* = 0.443; 95% CI = -1.000 to 0.438). Individuals receiving algorithmic advice reported higher initial confidence ($t = 3.93$; *P* < 0.001, 95% CI = 0.149 to 0.050) and spent less time on a problem ($t = 2.00$; *P* = 0.045; 95% CI = 0.00076 to 0.07293) when we analyzed all questions. However, if we compare confidence for only the first question subjects saw (before they received any advice), the difference in initial confidence is not significant (*t* $= - 0.20$; *P* = 0.403; 95% CI = − 0.203 to 0.082). The difference in time spent on a problem is also not significant when looking at only the first question ($t = 0.054$; *P* = 0.586; 95% CI = − 0.084 to 0.149). These results indicate that subjects were effectively equivalent in both conditions, as expected from random assignment. In the aggregate, when they received algorithmic advice, subjects became more confident in their initial guesses and spent less time on a problem in later questions.

### Main analyses

To test the preregistered hypotheses, we fit a series of mixed effects linear regressions with random slopes for each subject. The regression results are given in Table S2.

**Figure 1:**
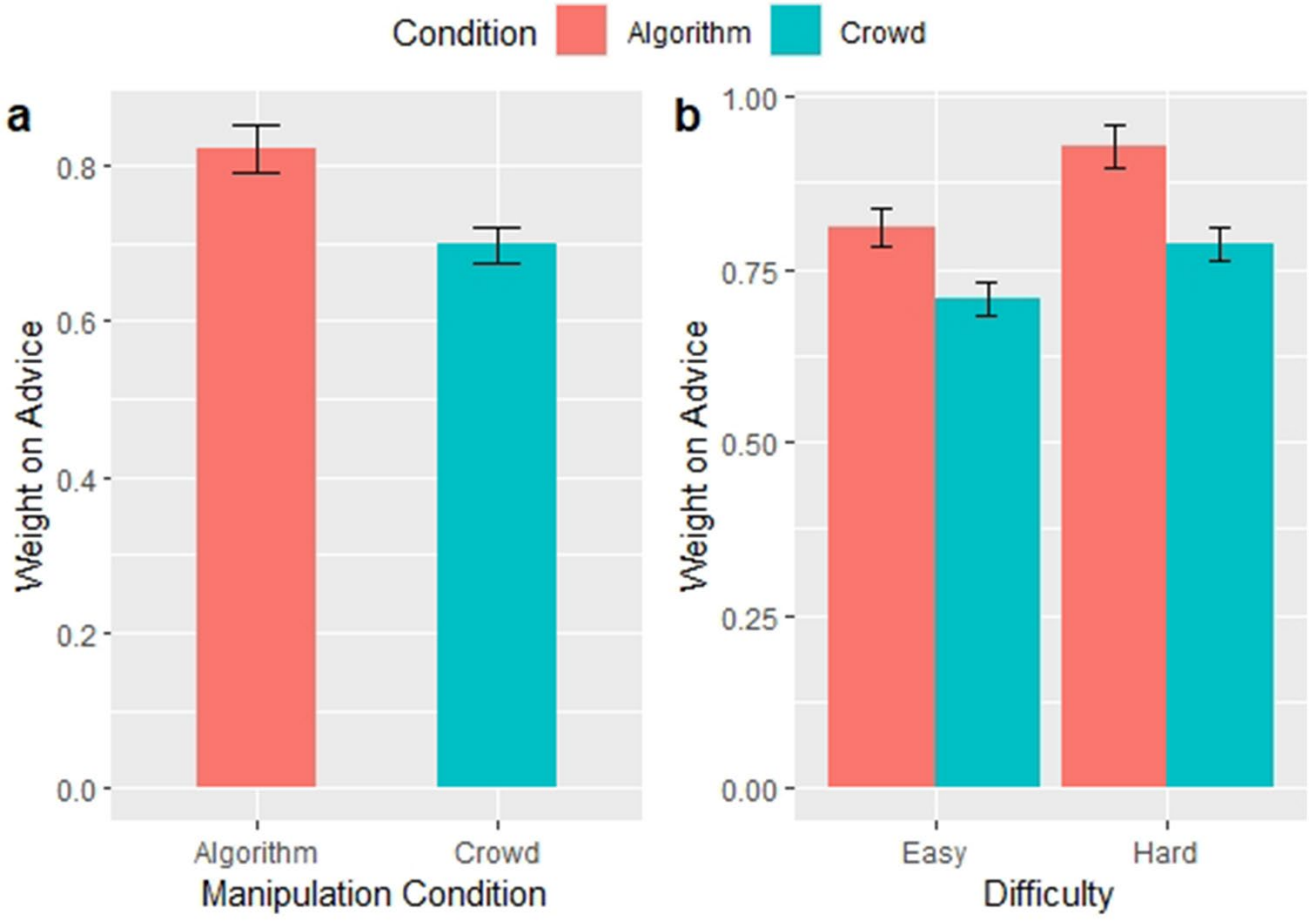
Source of advice affects subject weight on advice (Experiment 1). Each bar chart depicts results of the mixed effects regression model on N = 5083 observations. All models include accuracy as a control. Error bars correspond to the standard error of the estimated effect. (**a**) shows the main effect of advice source on WOA; the difference across conditions is significant (*p* < 0.001). (**b**) shows the effect of advice source on WOA across levels of difficulty; all differences are significant (*p* < 0.05). Panel A shows the effects using Model 1 from Table S2, Panel B shows the effects using Model 2 from Table S2.

Effect sizes and confidence intervals are shown for the effect of algorithmic advice and difficulty in Fig. 1 below. All figures were made using ggplot2^32^, version 3.3.

There is a positive and significant main effect of algorithmic advice on WOA (*B* = 0.108; *P* < 0.001; 95% CI = 0.058 to 0.158). Similarly, we find a positive and significant interaction effect of algorithmic advice and difficulty on WOA (*B* = 0.036; *P* = 0.029; 95% CI = 0.004 to 0.068)). That is, subjects who receive advice from algorithms on easy problems will revise their responses 11% more than subjects receiving advice from the crowd. Further, if a problem is difficult, subjects revised their answers by an additional 3.6% more when they receive advice from an algorithm, indicating that subjects rely even more on algorithms than they do on the advice of a crowd when the task is difficult.

Finally, we checked whether highly accurate subjects were disproportionately relying on algorithmic advice and found there was no significant difference (*B* = − 0.007, *P* = 0.81, 95% CI = − 0.067 to 0.053). We did not hypothesize this in our preregistration for the first experiment, although we investigated this further in experiments two and three.

### Experiment 2: Advice source as within-subjects treatment

In the second experiment we again show subjects pictures of human crowds and ask them to guess how many people are in the picture. However, in experiment two we make advice source a within-subjects condition. We do so because within-subject designs better control for any differences among subjects^33^. Subjects received five questions for which they received advice that was labeled as the average of 5000 human guesses, and five questions for which they received advice that was labeled as being from an algorithm trained on 5000 pictures. We also introduced numeracy as a new control variable in this experiment^34^. The second experiment includes 514 people, after following the same exclusion procedures for the first experiment, with the exception of the manipulation check, which we did not use because the advice condition was within-subjects.

**Figure 2:**
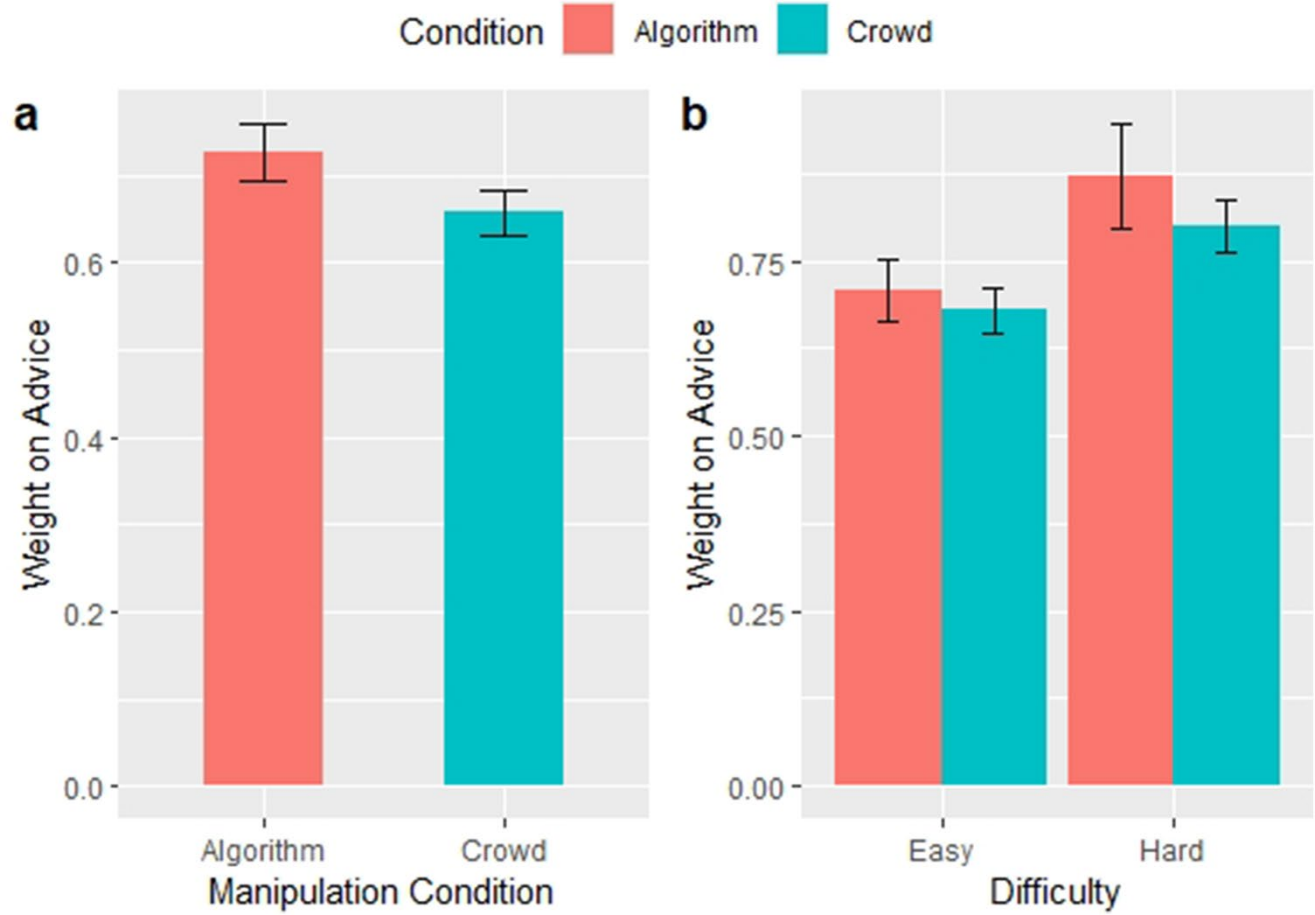
Source of advice affects subject weight on advice (Experiment 2). Each bar chart depicts results of the mixed effects regression model on N = 4,905 observations. All models include accuracy as a control. Error bars correspond to the standard error of the estimated effect. (**a**) shows the main effect of advice source on WOA; the difference across conditions is significant (*P* < 0.001). (**b**) shows the effect of advice source on WOA across levels of difficulty; all differences are significant (*P* < 0.05). Panel A is for Model 1 in Table S3, Panel B is for Model 2.

Results from the second experiment reinforced the results from the first experiment. In the baseline model without interactions, subjects relied more strongly on advice when it was labeled as algorithmic (*B* = 0.069; *P* < 0.001; 95% CI = 0.052 to 0.086). When interactions are analyzed however, the main effect of algorithmic advice becomes non-significant (*B* = 0.027; *P* = 0.18; 95% CI = -0.013 to 0.0670). We also found that participants relied more on algorithmic than crowd advice for difficult questions (*B* = 0.038; *P* = 0.037; 95% CI = 0.002 to 0.074). The results indicate that there is a net effect of algorithmic appreciation, but that a positive impact is driven entirely by a reliance on algorithms for hard problems. The effects and associated standard errors can be seen in Fig. 2.

Finally, more accurate subjects relied on algorithmic advice to the same degree as less accurate subjects (*B* = 0.045; *P* = 0.15, 95% CI = − 0.017 to 0.107).

### Experiment 3: Incorporating low-quality advice

**Figure 3:**
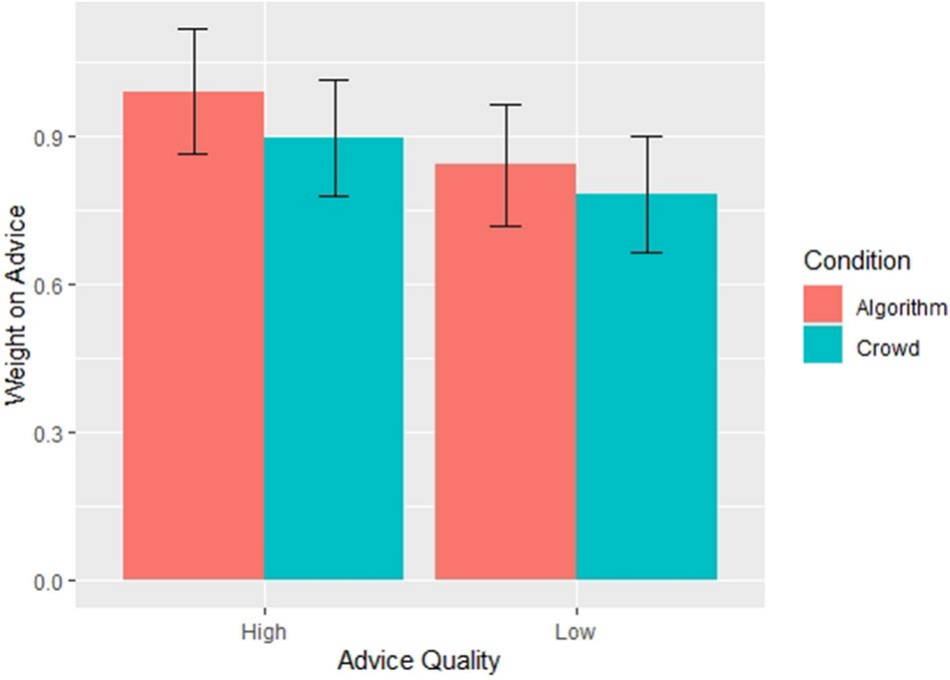
Source of advice affects subject weight on advice (Experiment 3). Each bar chart depicts results of the mixed effects regression model on N = 4365 observations. The exact model used is Model 3 in Table S4. Error bars correspond to the standard error of the estimated effect.

In the third experiment, we relax a significant assumption made in the other two experiments, in which the advice provided was always the correct answer, and thus was strictly high-quality advice. In the third experiment, we introduce low quality advice, to test whether the findings relied on providing subjects with high quality advice. Low quality advice was a within-subjects condition such that all participants saw the correct answer as advice for half of the questions, and advice that was 100% too high for the other half of the questions. The choice of 100% too high was based on pilot that tested advice that was too high by 50%, 100%, and 150%. Experiment three reinforced the results from the first two experiments. We show the effects of quality and advice source below in Fig. 3—these effects are taken from Model 3 in Table S4.

Subjects relied more strongly on algorithmic advice (*B* = 0.059; *P* < 0.048; 95% CI = 0.0004 to 0.1180), and this effect was magnified for difficult tasks (*B* = 0.037; *P* = 0.028; 95% CI = 0.004 to 0.071). Subjects who were more accurate initially did not rely more on algorithmic advice than the advice of a crowd (*B* = 0.064; *P* = 0.052). Subjects relied more strongly on good advice than on bad advice (*B* = 0.11; *P* < 0.001, 95% CI = 0.084 to 0.144), and this effect was greater when the source was an algorithm (*B* = 0.035; *P* = 0.043; 95% CI = 0.001 to 0.068). Another way to interpret this finding is that subjects penalized algorithms more for providing bad advice. When a crowd of peers provided low quality advice compared to high quality advice, the baseline from experiments one and two, subjects exhibited a WOA of 11% lower, while bad advice from an algorithm reduced WOA by more than 14%. Lastly, the effect of bad advice was moderated by the difficulty of the question (*B* = − 0.146; *P* < 0.001; 95% CI = − 0.181 to − 0.111). What this means in light of the research question is more nuanced. Our research question is whether people rely more on algorithmic advice than social advice when intellective tasks become harder, and whether advice quality moderates that effect. The interaction of advice quality and question difficulty may not specifically answer that question, but what it does tell us is that subjects are sensitive to both difficulty and quality in tandem, even after accounting for other factors. Further, we find that our primary treatment—advice source—has a significant effect on WOA even after including this interaction. This result suggests that source has a robust effect across combinations of conditions, lending additional support to one of our main claims.

### Additional analyses and robustness checks

It is possible that the findings are due to some unobservable individual skill or quality not eliminated by random assignment. Consequently, we conducted an analysis of covariance^35^ to predict WOA and change in confidence using initial accuracy, initial confidence, and initial time spent on the task across each level of advice source and difficulty. Thus, we are able to determine the effect of advice source and difficulty on WOA after controlling for differences in individuals’ skill (accuracy), perceived skill (confidence), and effort (time).

**Figure 4:**
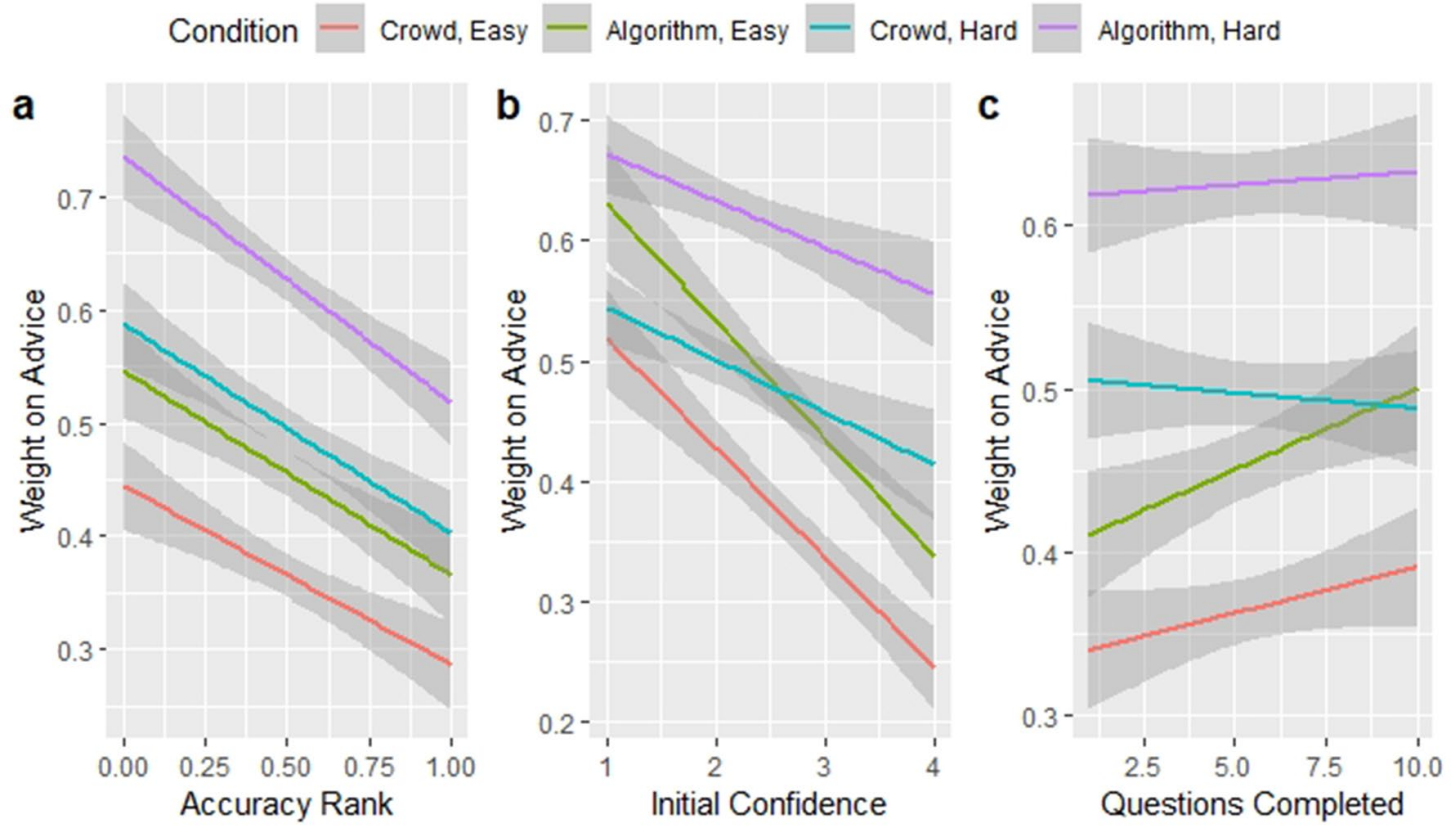
Effects of accuracy, initial confidence, and initial time. Each plot depicts a linear regression using a control variable to explain WOA delineated by advice condition and difficulty (N = 1249). The shaded areas depict 95% confidence intervals. WOA is regressed on (Panel **A**) initial accuracy, (Panel **B**) initial confidence, and (Panel **C**) the number of questions a subject has completed thus far.

Across all levels of accuracy, initial confidence, and initial time, subjects consistently exhibited higher WOA when receiving advice from an algorithm, when comparing hard questions to hard questions and easy questions to easy questions, see Fig. 4 below. This combination of algorithmic advice and problem difficulty creates the most significant change in subject estimates, with virtually no overlap of the 95% confidence intervals.

Finally, we conducted robustness checks on the main models (Fig. S2). We removed subsets of the data to ensure extreme values were not adversely impacting the findings. We excluded the top and bottom 2.5% responses for confidence, time per question, and overall time spent. Across all alternative regressions the findings are consistent. To check for multicollinearity, we removed control variables stepwise. Removing accuracy, initial confidence, and both accuracy and initial confidence did not change the results.

## Summary of experimental results

When comparing effects across all three experiments, there is remarkable consistency in the most important finding, namely, people rely more on algorithmic advice than crowd advice as tasks become more difficult. When using the baseline model outlined in the Analytical Approach in the following section, we find no significant differences in the interaction between algorithmic advice and question difficulty. For all three experiments, the effect is between 0.035 and 0.038, indicating that people rely substantially more on algorithmic advice for difficult questions than for easy questions, even after accounting for numeracy, accuracy, confidence, advice quality, and the number of prior questions answered. A summary of the results in each experiment in Table 1.

**Table 1:** Summary of Experimental Findings.

Claim	Experimental support		
	1	2	3
Subjects will rely more on algorithmic advice than equally good human advice	Yes	Partial*	Yes
Subjects receiving algorithmic advice will rely more on advice in difficult questions	Yes	Yes	Yes
Highly accurate subjects will rely more on algorithmic advice than inaccurate subjects**	No	No	No
Bad advice from an algorithm more strongly reduces weight on advice than bad advice from a crowd	N/A	N/A	Yes

*supported in baseline model without interactions and controls.

**This hypothesis was preregistered only for Experiment 3. When using an alternative measure of accuracy that allowed for significant outliers, we observed a positive and significant effect of the interaction between accuracy and algorithmic advice in both Experiment 1 and 2, so we preregistered a hypothesis about this effect for Experiment 3. We then observed that the observed post-hoc effect in Experiment 1 and 2 were due to outliers. We thus changed the accuracy measure to be the percentile rank of accuracy on a question, to prevent outliers from strongly influencing results.

## Discussion

These three experiments contribute to the burgeoning literature on social influence, the wisdom of the crowds^21,22,36^, and the role of algorithms in decision making. We provide large-sample experimental evidence that for intellective tasks, humans are more accepting of algorithmic advice relative to the consensus estimates of a crowd, echoing the results of prior literature^12^. Most importantly, we found that subjects exhibit greater algorithmic appreciation as intellective tasks became more difficult. With difficult intellective tasks, there is a robust and practically significant impact of algorithmic appreciation.

Other findings using experiments and the Judge Advisor System have found that the difficulty of a task had no effect on algorithmic appreciation^12^, or that as tasks became more difficult humans would rely less on algorithms^19^. Our paper finds the opposite, while more strongly and precisely manipulating difficulty, in an environment with incentives to do well, while controlling for the accuracy of subjects, whether the advice was within or between subjects, the quality of the advice, the numeracy of the subjects, and the confidence of the subject.

Humans may show a preference toward algorithmic advice depending on how close their initial guess is to the advice they receive^19^. Those with a history of recent accuracy may strongly discount the advice of others, while incorporating the advice of an algorithm, because if people perceive they are good at intellective tasks then they likely question why they should accept the advice of the less skilled crowd^19^. Thus, we expected that highly accurate individuals would demonstrate algorithmic appreciation more than the less accurate; however, our experiments did not support this claim.

Humans can discriminate between good and bad advice, and rely less on low-quality advice than they do on high-quality advice^37^. However, the interaction between advice quality and whether the advice comes from an algorithm or group of other humans is largely ignored—humans might respond differently to algorithmic mistakes compared to mistakes from a wise crowd when a question is easy or hard. We build on prior research that examines algorithmic advice-taking^19,38^ and advice quality by introducing a reference group, the advice of a crowd, with equally good (bad) advice. We tested whether low-quality advice from algorithms creates a stronger negative effect than low-quality advice from humans. Our experimental results suggest that when advice quality deteriorates (i.e., goes from high to low), algorithms will be penalized to a greater degree than a crowd of advisors.

An important feature of our experiment is the choice of reference group relative to algorithmic advice. Large, dispersed human crowds have both historically made accurate guesses^24,25^ and people strongly respond to the wisdom of the crowd^26^. Indeed, we observed that subjects who received advice from the crowd significantly revised their answers. However, the recommendation of an algorithm still had a stronger effect, across multiple specifications and experimental conditions. Thus, we argue that simply labeling advice as “algorithmic” or derived from machine learning can cause a meaningful shift in human behavior. We used a relatively weak manipulation –simply changing the label of the advice as either algorithmic or the average of a crowd. The consistent, statistically robust differences observed by changing only a few words demonstrate that these effects are strong.

The study has some limitations. The subjects recruited might have been more comfortable with technology and thus had a higher propensity towards algorithmic advice than the larger public. However, even if the subjects demonstrate more algorithmic appreciation than the public overall, we expect that the shift towards algorithmic advice for difficult, intellective tasks is a universal effect. Further, as experiment two demonstrates, there is equal appreciation for crowd and algorithmic advice when completing easy tasks. It is also possible that this task, which is relatively mundane and tedious, may have unique characteristics that cause people to lean disproportionately on algorithmic advice as difficulty increases. Specifically, for intellective tasks, people may be more likely to rely on algorithmic recommendations, whereas for tasks that have significant negotiation or generative components, which require subjective judgments, people may feel less comfortable relying on algorithms entirely. However, we leave these alternative task types to future research.

Governments and corporations have a strong interest in leveraging AI. This can be at the expense of consumers and citizens, who may not know that their data are harvested, stored, and analyzed. People whose data are used to calibrate algorithms could be affected by them, positively or negatively, by social or corporate policies based on AI. The public seeks interventions that solve important societal problems, such as income inequality, medical research, or systemic biases in institutions. Because interventions can be harmful, carefully managed research, followed by trials, is necessary to minimize unintended effects. If governments wish to spend citizens’ taxes wisely, we need them to take an evidence-based approach to social policy, with AI as a potential research methodology. Citizens need to be engaged by freely sharing data that might address private matters, such as spending patterns when evaluating the potential outcomes of universal basic income. There is an inherent trade-off in evidence-based public decision making in that some proportion of the population need to take a health, privacy, or other risk to support societal goals. Further research should investigate how improving predictive capabilities can be responsibly leveraged across government and private enterprises.

As tasks become more complex and data intensive algorithms will continue to be leveraged for decision making. Already, algorithms are used for difficult tasks such as medical diagnoses^39^, bail decisions^40^, stock picking^41^, and determining the veracity of content on social media^42^. The findings reveal a reliance on algorithms for difficult tasks and it is important for decision-makers to be vigilant in how they incorporate algorithmic advice, particularly because they are likely predisposed towards leaning on it for difficult, thorny problems. While algorithms can generally be very accurate, there are instances of algorithms quietly making sexist hiring decisions in one of the largest companies in the United States^43^, initiating plane crashes^44^, or causing racist bail decisions^45^. Consequently, individuals and organizations leveraging big data to make decisions must be cognizant of the potential biases that accompany algorithmic recommendations, particularly for difficult problems. Decision makers should remember that they are likely to rely more on algorithms for harder questions, which may lead to flawed, biased, or inaccurate results. Accordingly, extreme algorithmic appreciation can lead to not only complacency, but also ineffective policies, poor business decisions, or propagation of biases.

## Methods

This study was approved by the University of Georgia Institutional Review Board, project 00001012. Subjects gave written informed consent both before and after participation in the study. All methods were carried out in accordance with relevant guidelines and regulations. We conducted three preregistered experiments to test the conditions under which humans accept advice. Following a Judge Advisor System approach^30^, subjects were asked to answer a question, then were exposed to advice, and then asked to submit a second answer. The links to our preregistrations for experiment 1, experiment 2, and experiment 3 are: https://osf.io/ym3ug, https://osf.io/hyz6d, and https://osf.io/vgh9k.

### Subjects

For experiment 1, we conducted a power analysis that indicated we needed 235 subjects per group. With two groups that is 470 subjects. We used a t-test for evaluating the difference between two independent means using the statistical software G Power^46^. We conducted a two tailed test, with an effect size of 0.3, an error probability of 0.05, power of 0.90, and an allocation ratio of 1. Subjects were recruited from Amazon Mechanical Turk (AMT). We started with 611 respondents recruited from AMT. Of those, 16 were duplicate IP addresses, 27 failed the attention check, 3 did not consent to their data being used, and 21 failed the manipulation check. Lastly, we excluded 14 subjects who had no deviation in their weight on advice, i.e. subjects who always either took the advice perfectly or who always completely ignored the advice. The analysis is based on the 530 remaining subjects, compared with the preregistered plan of 470 subjects. We oversampled because we did not know a priori how many subjects would be excluded. As part of our robustness checks we removed subjects based on time spent on a problem and confidence. The findings did not meaningfully change. Each subject was paid USD 1.50 to complete the experiment, and an additional bonus of USD 0.50 was given to subjects in the top 20% of accuracy in their final answers. Subjects were aware that a bonus was available for the most accurate respondents, but were not told the exact amount of the bonus, following prior usage of bonuses in online experiments^36^.

For experiments two and three we followed a similar approach, again recruiting subjects from Amazon Mechanical Turk. Subjects who participated in one of the experiments were not allowed to participate in a subsequent experiment, because we wanted to obtain as large a cross-section of the population as possible, and because we informed subjects of the experimental manipulation after the experiment was completed. We review the details of how we excluded subjects for experiments two and three in the Supplementary Information.

### Task

All subjects saw ten images with crowds of between 15 (for the easiest question) and 5000 (for the hardest question) humans. Easier questions were either the bottom left or bottom right quadrant of a harder image and were zoomed in so that each picture was the same size. The pictures were from an annotated dataset with professional assessments of the number of people in a picture^47^. For each picture, a subject submitted an initial guess, along with their confidence. Subjects were then given advice and asked to resubmit an estimate along with their new level of confidence. Each subject saw ten pictures, five easy and five hard, which vary by the number of people pictured. The difficulty manipulation was within-subjects—all respondents saw the same questions. The type of advice was between subjects. Each subject was placed in one of two groups—one received advice described as “an algorithm trained on 5000 images” and one received advice described as “the average of 5000 other people”. To control for advice quality, which is known to affect advice discounting^16^, the advice was always the correct number of people in an image, as reported in the image database. We later manipulate advice quality in experiment three.

Subjects were reminded of their prior answer when answering the question the second time. Subjects answered how confident they were in both the initial and subsequent guess. Easier questions are subsets of harder questions—for each picture the easier version of the question was always the bottom left or bottom right quadrant of the harder picture. We bolded the source of the advice, which was described as either “an algorithm trained on 5000 images similar to this one” or “the average guess 5000 other people”. In experiment 1 the source of the advice was between subjects, and thus never changed for a subject. In experiment two we relaxed this assumption and showed advice as within-subjects. Question order was randomized, so that subjects could see easy or hard questions in any order, but subjects always saw the Post question directly after the Initial Question.

### Analytical approach

We used multilevel mixed-effects linear regression with random intercepts—fit using the lme4 package, version 1.1.23, in the R computing environment ^48^—to analyze the effects of the advice type and task difficulty on weight on advice, time, and confidence. We control for both the initial confidence in an estimate prior to seeing advice, and for accuracy prior to advice. Our main model is:

$$y_{ik} = \beta_{0i} + \beta_{1} {\text{AlgoCondition}}_{i} + \beta_{2} {\text{Difficulty}}_{k} + \beta_{3} {\text{AlgoCondition}}_{i} \times {\text{ Difficulty}}_{k} + \beta X_{ik} + \varepsilon_{ik}$$

Here, $y_{ik}$ is one of the dependent variables for participant $i$ and problem $k$; $\beta_{0i}$ is the slope for participant $i$; ${\text{AlgoCondition}}_{i}$ and ${\text{Difficulty}}_{k}$ are categorical variables indicating the advice condition and problem difficulty respectively; and $X_{ik}$ is a vector of control variables. For experiment three we added categorical variables for quality of advice and the interaction between quality of advice and algorithmic advice. We also added variables for accuracy of an estimate prior to advice being given, and the interaction of accuracy with algorithmic advice. We tested for the appropriateness of using a linear mixed effects model by plotting the standardized residuals against the standard normal distribution, see Fig. S3 in the Supplemental Information.

### Dependent variable

*Weight on Advice (WOA)*: The formula for WOA is $WOA_{ik} = { }\frac{{\left| {final{ }\;estimate_{ik} - initial\;estimate_{ik} } \right|}}{{\left| {recommendation_{k} - initial\;estimate_{ik} } \right|}}$. A WOA of one means an individual changed their answer to equal the advice given. A WOA of zero means an individual did not change their answer at all after receiving advice, and a WOA of 0.5 means an individual took the average of the advice given and their initial answer. According to recommended practices, we drop observations where the initial estimate is equal to the recommendation. We excluded observations where WOA was greater than two and less than negative one ^20^.

### Independent variables

*Difficulty*: Categorical variable representing whether an image was easy or hard. Hard images coded as 1.

*Algorithmic Advice*: Categorical variable representing whether a subject received algorithmic advice for that question. Algorithmic advice coded as 1.

*Accuracy*: To control for skill in estimating crowd size, we calculate a subject’s relative question-level accuracy as follows:

$$Error_{ik} = \left| {Initial{ }Answer_{ik} - Correct{ }Answer_{k} } \right|/Correct{ }Answer_{k}$$.

To improve interpretability, we take the inverse of a subject’s error: $Accuracy_{ik} = Error_{ik}^{ - 1}$. Thus, subjects who are more accurate had lower error estimates. To control for outliers, we then transform each subject’s accuracy into the percentile rank for that question.

*Advice Quality*: Dummy variable representing whether advice was accurate or inaccurate. Inaccurate advice was 100% too high. Accurate advice coded as 1.

### Control variables

*Initial Confidence*: a subject’s response to the question “How confident are you that your answer is within 10% of the true answer?” prior to receiving advice. 1 = Not at all confident, 2 = Not very confident. 3 = Somewhat confident. 4 = Extremely confident.

*Round Number:* Because questions were in a random order, this variable described how many questions a subject had worked on thus far. Ranges from one to ten.

*Numeracy:* A measure to determine how well a subject understands fractions, decimals, and other numbers, previously used to establish numeracy in assessments of algorithmic advice taking^12^ and medical decisions ^34^. Ranges from one to eleven.

## Supplementary Information

Supplementary Information

## Data Availability

The datasets generated and analyzed during the current study are available in the Open Science Foundation repository: experiment 1, experiment 2, and experiment 3.
