# Supplementary material for: Humans rely more on algorithms than social influence as a task becomes more difficult
Source: Sci Rep. 2021 Apr 13;11:8028. doi: 10.1038/s41598-021-87480-9 (PMC8044128; doi:10.1038/s41598-021-87480-9)
Supplement: Supplementary file 1 — Supplementary Information [file 41598_2021_87480_MOESM1_ESM.pdf]

Supplementary Information

Humans Rely More on Algorithms than Social Influence as a Task Becomes More Difficult

Eric Bogert[1,*], Aaron Schecter[1], Rick Watson[1]

[1]Management Information Systems Department, University of Georgia, Athens, GA, USA

*Correspondence to etbogert@uga.edu

Table S1. Summary Statistics of Experiment 1

Variable	Experiment 1		Experiment 2		Experiment 3	
	Mean	SD	Mean	SD	Mean	SD
WOA	0.487	0.370	0.525	0.367	0.451	0.381
Change in Confidence	0.178	0.440	0.192	0.453	0.116	0.380
Change in Time	-0.146	1.212	0.068	2.720	0.063	10.04
Initial Accuracy	0.500	0.287	0.501	0.288	0.500	0.287
Initial Confidence	2.438	0.904	2.637	0.823	2.696	0.826

Table S2. Model Results predicting Weight on Advice (Experiment 1: Algorithmic Advice Between-Subjects).

Variable	Model 1	Model 2	Model 3
Intercept	0.455 ***	0.466 ***	0.683 ***
	(0.016)	(0.018)	(0.026)
Algorithmic Advice	0.114 ***	0.094 ***	0.108 ***
	(0.018)	(0.026)	(0.025)
Difficulty	0.150 ***	0.132 ***	0.081 ***
	(0.008)	(0.012)	(0.012)
Initial Accuracy	-0.203 ***	-0.205 ***	-0.202 ***
	(0.016)	(0.022)	(0.022)
Algorithmic Advice * Difficulty		0.037 *	0.036 *
		(0.017)	(0.017)
Algorithmic Advice * Initial Accuracy		0.002	-0.007
		(0.031)	(0.031)
Initial Confidence			0.003 *
			(0.001)
Round Number			-0.088 ***
			(0.006)
Observations	5083	5083	5083
AIC	3043.487	3054.017	2892.011

The dependent variable is Weight on Advice. Initial Accuracy is the percentile rank score (0.00 to 1.0) of how accurate a subject's first guess was, relative to other subjects for that question. Initial confidence is on a Likert scale from 1-4. Algorithmic Advice is a categorical variable (algorithmic condition = 1, human advice = 0) and Difficulty is an ordinal variable (hard questions = 1, easy questions = 0). Standard errors are in parentheses. There were N = 530 subjects in Experiment 1.

*** $p < 0.001$; ** $p<0.01$; * $p<0.05$.

Table S3. Model Results Predicting Weight on Advice (Experiment 2: Algorithmic Advice Within-Subjects).

Variable	Model 1	Model 2	Model 3
Intercept	0.444 ***	0.465 ***	0.730 ***
	(0.014)	(0.017)	(0.054)
Algorithmic Advice	0.069 ***	0.027	0.027
	(0.009)	(0.020)	(0.020)
Difficulty	0.139 ***	0.120 ***	0.089 ***
	(0.009)	(0.013)	(0.014)
Initial Accuracy	-0.050 **	-0.072 **	-0.069 **
	(0.016)	(0.023)	(0.023)
Algorithmic Advice * Difficulty		0.039 *	0.038 *
		(0.018)	(0.018)
Algorithmic Advice * Initial Accuracy		0.045	0.045
		(0.032)	(0.031)
Initial Confidence			-0.043 ***
			(0.008)
Numeracy			-0.014 **
			(0.005)
Round Number			-0.003
			(0.002)
Observations	4905	4905	4905
AIC	3110.667	3119.503	3111.134

The dependent variable is Weight on Advice. Initial Accuracy is the percentile rank score (0.00 to 1.0) of how accurate a subject's first guess was, relative to other subjects for that question. Initial confidence is on a Likert scale from 1-4. Algorithmic Advice is a categorical variable (algorithmic condition = 1, human advice = 0) and Difficulty is a categorical variable (hard questions = 1, easy questions = 0). Standard errors are in parentheses. There were N = 514 subjects in Experiment 2.

*** $P < 0.001$; ** $P < 0.01$; * $P < 0.05$

Table S4. Model Results predicting Weight on Advice (Experiment 3: Including Low-Quality Advice).

Variable	Model 1	Model 2	Model 3
Intercept	0.329 ***	0.609 ***	0.865 ***
	(0.019)	(0.034)	(0.059)
Algorithmic Advice	0.135 ***	0.092 **	0.059 *
	(0.023)	(0.030)	(0.030)
Difficulty	0.160 ***	0.098 ***	0.167 ***
	(0.009)	(0.013)	(0.016)
Initial Accuracy	-0.052 **	-0.080 ***	-0.080 ***
	(0.017)	(0.023)	(0.023)
Initial Confidence		-0.071 ***	-0.074 ***
		(0.008)	(0.008)
Round Number		-0.008 ***	-0.008 ***
		(0.002)	(0.001)
Algorithmic Advice * Difficulty		0.035 *	0.037 *
		(0.017)	(0.017)
Algorithmic Advice * Initial Accuracy		0.058	0.063
		(0.033)	(0.033)
Quality			0.114 ***
			(0.015)
Numeracy			-0.036 ***
			(0.005)
Algorithmic Advice * Quality			0.035 *
			(0.017)
Difficulty * Advice Quality			-0.146 ***
			(0.018)
Observations	4365	4365	4365
AIC	2459.132	2390.082	2272.17

The dependent variable is Weight on Advice. Initial Accuracy is the percentile rank score (0.00 to 1.0) of how accurate a subject's first guess was, relative to other subjects for that question. Initial confidence is on a Likert scale from 1-4. Algorithmic Advice is a categorical variable (algorithmic condition = 1, human advice = 0) and Difficulty is a categorical variable (hard questions = 1, easy questions = 0). Standard errors are in parentheses. There were N = 456 subjects in Experiment 3.

*** $P < 0.001$; ** $P < 0.01$; * $P < 0.05$

1 PREREGISTERED HYPOTHESES AND ANALYSES WITH ALTERNATE DEPENDENT VARIABLES

In addition to studying how people responded behaviorally by their weight on advice, we preregistered hypotheses related to how people responded cognitively, both through the time they spent on a question and their self-reported confidence in their answers. This allowed us to study both behavioral (weight on advice) and cognitive (confidence and time spent on a problem) manifestations of reliance on machine intelligence relative to social influence. We chose to report these results on alternate dependent variables in the supplementary information to increase the readability and decrease the length of the main paper.

We hypothesized that subjects would demonstrate greater reliance on machine intelligence than social influence, and most importantly, that effect would be stronger in more difficult tasks. For time-related measurements this would manifest through subjects spending less time when they are advised by an algorithm, and that this effect would be stronger in more difficult tasks. Using time as a measure of cognitive dependence is a previously established measure of cognitive effort (1). For confidence-related measurements this would result in subjects becoming more confident, based on a measure using a Likert scale, when receiving algorithmic advice, and this effect being stronger in more difficult tasks.

For experiment 1 and 2, our hypotheses were as follows.

Table S5. Preregistered Hypotheses for Experiment 1 and Experiment 2

#	Hypothesis
H1a	The effect of algorithmic advice on weight on advice will be greater than the advice of a crowd.
H1b	The effect of algorithmic advice on weight on advice will be stronger for a more difficult problem.
H2a	Algorithmic advice will result in less time spent determining an answer relative to advice from a crowd.
H2b	The effect of algorithmic advice on time, relative to advice from a crowd, will be stronger for a more difficult problem.
H3a	Algorithmic advice makes humans more confident in decisions than the advice of crowds.
H3b	The effect of algorithmic advice on decision confidence will be stronger for a more difficult problem.

In experiment three we introduced low-quality advice, and added several hypotheses related to those effects. Those additional hypotheses are listed below. Because we preregistered these hypotheses we include them here, because it is a best practice of preregistered research to include all hypotheses from a preregistration (2). We believe the most interesting hypothesized effects are for Hypothesis 4 and Hypothesis 5b because those effects are related to algorithmic advice.

Table S6. Additional Preregistered Hypotheses for Experiment 3

#	Hypothesis
H4	Subjects who are more skilled at a task will rely more strongly on algorithmic advice than advice from a crowd.
H5a	Low quality advice reduces future reliance on the advice source
H5b	Low quality advice will more strongly reduce reliance on algorithmic advice than reliance on crowd advice
H5c	Low quality advice will more strongly reduce reliance on advice for easy questions than hard questions.

These hypotheses directly measured effects related to machine intelligence and social influence, whereas Hypothesis 5a and Hypothesis 5c did not hypothesize about differences between machine intelligence and social influence. Thus, Hypothesis 5a and 5c are more applicable to general behavior and decision-making literature, whereas Hypothesis 4 and 5b are directly applicable to the burgeoning literature on algorithmic appreciation.

2 RESULTS

2.1 Experiment 1:

Increase in confidence is higher when receiving advice from an algorithm and increases with task difficulty

We found that algorithmic advice increased confidence (*B* = 0.043; *P* = 0.046; 95% Confidence Interval (CI) = 0.002 to 0.083), supporting H3a and in line with the effect we observed on weight on advice. Furthermore, the interaction between algorithmic advice and difficulty was significant and positive (*B* = 0.053; *P* = 0.003; CI = 0.022 to 0.093), supporting H3b and in line with the effect we observed on weight on advice. When individuals receive advice from an algorithm rather than a crowd for a hard task, they are 5.3% more confident in their final answer compared to their initial guess. Overall, the results suggest that when subjects receive advice from an algorithm rather than a purported crowd of other humans, they become more confident in their answers, and this effect is stronger as tasks become more difficult. Contrary to our hypotheses, these results were not observed when we studied time as a dependent variable, indicating no support for H2a and H2b. Results are in Table S7 below.

Table S7: Experiment 1 Analyses on Alternative DVs

	Change in Confidence DV			Change in Time DV		
**Variable**	**Model 1**	**Model 2**	**Model 3**	**Model 4**	**Model 5**	**Model 6**
Intercept	-0.035	0.001	0.777 ***	-0.097 *	-0.097	0.023
	(0.018)	(0.021)	(0.029)	(0.043)	(0.055)	(0.085)
Algorithmic Advice	0.043 *	-0.031	0.016	0.048	0.049	0.047
	(0.021)	(0.030)	(0.027)	(0.038)	(0.078)	(0.078)
Difficulty	0.092 ***	0.060 ***	-0.103 ***	0.263 ***	0.238 ***	0.236 ***
	(0.011)	(0.015)	(0.014)	(0.033)	(0.047)	(0.048)
Initial Accuracy	0.287 ***	0.246 ***	0.253 ***	-0.413 ***	-0.387 ***	-0.389 ***
	(0.020)	(0.028)	(0.024)	(0.059)	(0.083)	(0.083)
Algorithmic Advice * Difficulty		0.064 **	0.058 **		0.049	0.047
		(0.021)	(0.018)		(0.066)	(0.066)
Algorithmic Advice * Initial Accuracy		0.081 *	0.053		-0.053	-0.047
		(0.039)	(0.034)		(0.117)	(0.117)
Round Number			-0.002			-0.018 **
			(0.002)			(0.006)
Initial Confidence			-0.286 ***			-0.007
			(0.007)			(0.021)
Observations	5083	5083	5083	5083	5083	5083
AIC	5255.830	5257.079	3880.841	16279.534	16288.811	16296.853

*** p < 0.001; **p < 0.01; *p < 0.05.

The dependent variables are the percentile change in confidence (model 1, 2, and 3) and the percentile change in time spent on a problem (model 4, 5, and 6). Initial Accuracy is the percentile rank score (0.00 to 1.0) of how accurate a subject's first guess was, relative to other subjects for that question. Initial confidence is on a Likert scale from 1-4. Algorithmic Advice is a dummy variable (algorithmic condition = 1, human advice = 0) and Difficulty is a dummy variable (hard questions = 1, easy questions = 0). Standard errors are in parentheses. There were N = 530 subjects in Experiment 1.

2.1.1 Experiment 1: Robustness Checks

Finally, we conducted several robustness checks on our main models (model 3 and model 6 above). See Figure S1A for change in time spent on a problem and S1B for change in confidence. We removed subsets of our data to ensure extreme values were not adversely impacting our findings. The subgroups exclude the following people: the top 5% and bottom 5% and both top and bottom 2.5% in accuracy, and initial time spent on a question, and the top and bottom 2.5% in confidence on initial questions. To check for multicollinearity, we removed control variables step-wise.

Removing accuracy, initial confidence, and both accuracy and initial confidence did not change our results – all supported hypotheses remained supported at the 0.05 level, and all unsupported hypotheses remained unsupported.

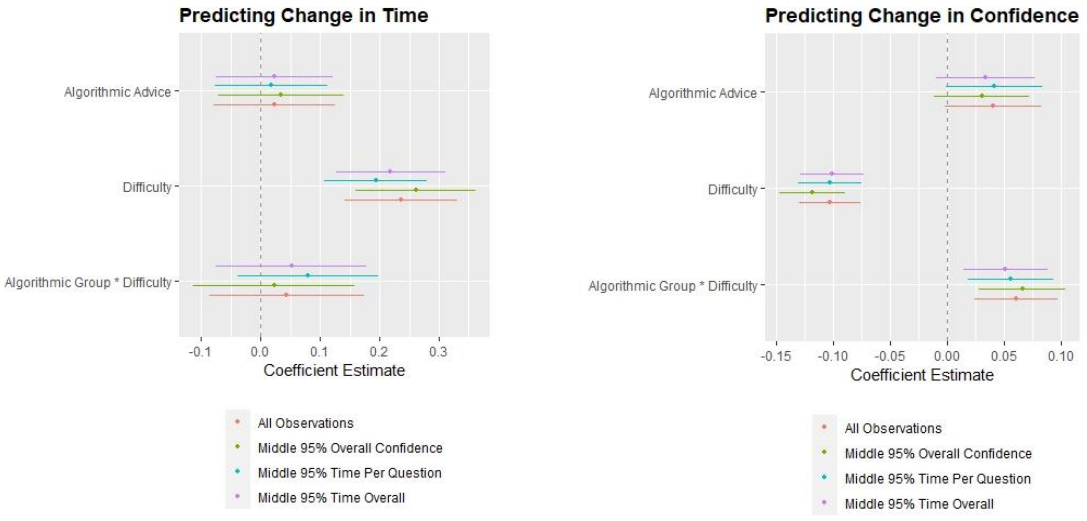

(A) (B)

Figure S1. Robustness checks for (A) change in time and (B) change in confidence across a variety of subsets.

We ran identical exclusions for WOA, our dependent variable from the main text. We removed subsets of our subjects to ensure our results were robust to outliers. Figure S2 shows that our effects are robust to removing these subsets.

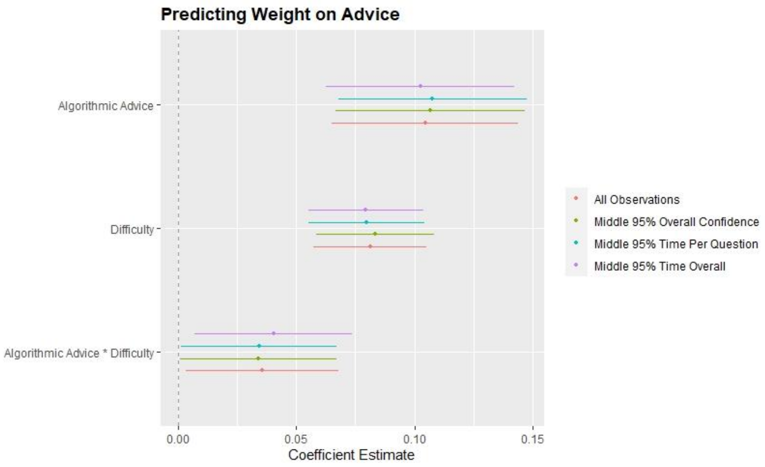

Figure S2. Robustness checks for Weight on Advice across subsets

2.2 Experiment 2

In experiment two, we followed the same procedure, except that subjects received advice as a within-subjects condition, resulting in each subject receiving advice labeled as algorithmic five times and advice labeled as the average of other humans five times. We used the same hypotheses from experiment one, and observed similar effects, shown in Table S8 below. Again, we observe that the effect of algorithmic advice on a change in confidence is positive and significant (*B* = 0.055; *P* < 0.001; 95% CI = 0.034 to 0.077), but that effect disappears after incorporating the interaction between algorithmic advice and difficulty, indicating partial support for H3a and support for H3b. We also observe

consistent effects related to time – subjects did not statistically significantly change the amount of time spent on a problem, regardless of advice source and whether we include interactions or other controls, indicating no support for H2a and H2b.

Table S8: Experiment 2 Analyses on Alternative DVs

	Change in Confidence DV			Change in Time DV		
**Variable**	**Model 1**	**Model 2**	**Model 3**	**Model 4**	**Model 5**	**Model 6**
Intercept	-0.071 ***	-0.053 **	1.084 ***	0.363 ***	0.374 **	0.717 ***
	(0.017)	(0.020)	(0.030)	(0.096)	(0.123)	(0.217)
Algorithmic Advice	0.055 ***	0.018	0.021	-0.114	-0.135	-0.137
	(0.011)	(0.026)	(0.021)	(0.077)	(0.175)	(0.175)
Difficulty	0.170 ***	0.135 ***	-0.132 ***	0.136	0.032	0.020
	(0.011)	(0.016)	(0.014)	(0.077)	(0.109)	(0.115)
Initial Accuracy	0.294 ***	0.294 ***	0.282 ***	-0.615 ***	-0.528 **	-0.515 **
	(0.021)	(0.029)	(0.024)	(0.135)	(0.191)	(0.191)
Algorithmic Advice * Difficulty		0.070 **	0.074 ***		0.207	0.202
		(0.023)	(0.019)		(0.155)	(0.155)
Algorithmic Advice * Initial Accuracy		0.001	0.003		-0.172	-0.166
		(0.040)	(0.032)		(0.269)	(0.269)
Round Number			0.000			-0.059 ***
			(0.002)			(0.013)
Initial Confidence			-0.378 ***			-0.007
			(0.008)			(0.054)
Observations	4905	4905	4905	4905	4905	4905
AIC	5247.113	5252.188	3385.244	23726.639	23731.135	23726.567

*** $p < 0.001$; ** $p<0.01$; * $p<0.05$.

The dependent variable is the percentile change in confidence (model 1, 2, and 3) and the percentile change in time spent on a problem (model 4, 5, and 6). Initial Accuracy is the percentile rank score (0.00 to 1.0) of how accurate a subject's first guess was, relative to other subjects for that question. Initial confidence is on a Likert scale from 1-4. Algorithmic Advice is a categorical variable (algorithmic condition = 1, human advice = 0) and Difficulty is a categorical variable (hard questions = 1, easy questions = 0). Standard errors are in parentheses. There were N = 514 subjects in Experiment 2.

2.3 Experiment 3

In experiment three we introduced low-quality advice and returned advice source to a between-subjects condition. We found that the effects related to confidence reinforced the results related to weight on advice. Subjects became more confident after receiving algorithmic advice relative to crowd advice, although this effect lost significance after controlling for advice quality (*B* = 0.020; *P* = 0.718; 95% CI = -0.038 to 0.069), demonstrating partial support for H1. Most importantly, subjects became more confident in difficult questions when receiving algorithmic advice, relative to advice of equal quality from a crowd, demonstrating support for hypothesis 2 (*B* = 0.067; *P* < .001; 95% CI = 0.032 to 0.102). Highly accurate subjects did not become statistically significantly more confident when receiving algorithmic advice relative to human advice (*B* = 0.066; *P* = 0.050; 95% CI = -0.00005 to 0.13185), although this effect is significant at a p value of 0.1. This indicates that we cannot reject the null hypothesis for hypothesis 4. Subjects became more confident when receiving high quality advice (*B* = 0.065; p < 0.001; 95% CI = 0.034 to 0.097), supporting hypothesis 5a. Unlike our results for weight on advice, subjects did not become significantly less confident when receiving low-quality algorithmic advice relative to low-quality crowd advice, as hypothesized in h5b (*B* = 0.014; *p* = 0.410; 95% CI = -0.020 to 0.050). Subjects also became significantly less confident when receiving low-quality advice for hard questions relative to easy questions, supporting hypothesis 5c (*B* = 0.049; *P* = 0.008; 95% CI = -0.085 to -0.013), demonstrating support for hypothesis 5c.

No hypotheses related to changes in time spent on a question were supported, regardless of whether we include only main effects used in our prior experiments (model 1), main effects plus the interaction between difficulty and algorithmic advice (model 2), or all interactions related to hypotheses plus main effects and controls (model 3).

Table S9: Experiment 3 Analyses on Alternative DVs

	Change in Confidence DV			Change in Time DV		
**Variable**	**Model 1**	**Model 2**	**Model 3**	**Model 4**	**Model 5**	**Model 6**
Intercept	-0.028	-0.012	1.019 ***	0.006	0.244	1.353
	(0.017)	(0.018)	(0.051)	(0.378)	-0.415	(1.171)
Algorithmic Advice	0.077 ***	0.048 *	0.010	0.418	-0.020	0.224
	(0.017)	(0.020)	(0.027)	(0.304)	-0.436	(0.750)
Difficulty	0.113 ***	0.084 ***	-0.073 ***	0.633 *	0.202	-0.202
	(0.010)	(0.015)	(0.017)	(0.304)	-0.433	(0.545)
Accuracy Rank	0.092 ***	0.090 ***	0.067 **	-0.961	-0.994	0.063
	(0.019)	(0.019)	(0.024)	(0.529)	-0.53	(0.752)
Algorithmic Advice * Difficulty		0.057 **	0.067 ***		0.850	0.829
		(0.021)	(0.018)		-0.609	(0.608)
Advice Quality			0.065 ***			-0.517
			(0.016)			(0.533)
Initial Confidence			-0.288 ***			0.130
			(0.008)			(0.204)
Numeracy			-0.021 ***			-0.230 **
			(0.004)			(0.079)
Round Number			-0.001			0.025
			(0.002)			(0.053)
Algorithmic Advice * Accuracy Rank			0.066			-1.462
			(0.034)			(1.059)
Algorithmic Advice * Advice Quality			0.015			0.877
			(0.018)			(0.607)
Difficulty * Quality		-	0.049 **			1.023
			(0.019)			(0.608)
N	4365	4365	4365	4365	4365	4365
AIC	3541.062	3541.574	2406.201	32532.609	32531.812	32532.145

*** p < 0.001; **p<0.01; *p<0.05.

The dependent variable is the percentile change in confidence (model 1, 2, and 3) and the percentile change in time spent on a problem (model 4, 5, and 6). Initial Accuracy is the percentile rank score (0.00 to 1.0) of how accurate a subject's first guess was, relative to other subjects for that question. Initial confidence is on a Likert scale from 1-4. Algorithmic Advice is a categorical variable (algorithmic condition = 1, human advice = 0) and Difficulty is a categorical variable (hard questions = 1, easy questions = 0). Standard errors are in parentheses. There were N = 456 subjects in Experiment 3.

2.4 Synthesized Results

In Table S10 we summarize results across each experiment and dependent variable. We combine H1A, H2A, and H3A into a single hypothesis, listed first in Table S10 below and combine H1B, H2B, and H3B into a single hypothesis, listed second in Table S10 below. We say a hypothesis was supported if, across all three experiments and all specifications of the experiments, we observed the relevant effect. We indicate partial support if, in at least one of the experiments, there was support for the hypothesis.

Table S10: Analyses on All Dependent Variables Across All Experiments

Hypothesis	Support (WOA)	Support (Time)	Support (Confidence)
Algorithmic advice will result in greater reliance on the advice than advice from a crowd	Yes	No	Partial
Algorithmic advice will be relied on more than advice from a crowd as task difficulty increases	Yes	No	Yes
Subjects who are more skilled at a task will rely more strongly on algorithmic advice than advice from a crowd.	No	No	No
Low quality advice reduces future reliance on the advice source*	Yes	No	Yes
Low quality advice will more strongly reduce reliance on algorithmic advice than reliance on crowd advice*	Yes	No	No
Low quality advice will more strongly reduce reliance on advice for easy questions than hard questions*	Yes	No	Yes

*Tested on experiment three exclusively

Overall, we observed that subjects became more confident when receiving algorithmic advice than when receiving crowd advice for difficult questions. This result provides further evidence that 1) the manipulation was salient, and 2) that participants relied meaningfully on the algorithmic advice to improve their answers. Clearly, people not only followed algorithmic advice, but felt better about themselves when they did so. Our null results related to time could be accounted for through several explanations. The most plausible is that time spent on a problem is not a manifestation of cognitive effort in the same way as weight on advice or scales related to confidence. Alternatively, this might be attributed to the fact that we recruited subjects from Amazon Mechanical Turk, resulting in our subjects being highly incentivized to work quickly, because they are paid per task rather than per unit of time.

2.5 Methods

2.5.1 Subjects:

We reviewed how we excluded subjects from experiment one in the main text of this article. In experiment two we used the same procedure for recruiting and excluding participants as we did in experiment one, with the additional criterion that we did not allow subjects from experiment one to participate. We started with data from 593 subjects. We oversampled slightly, relative to our stated sample size in the preregistration, because we did not know in advance how many subjects would fail our exclusion criteria. All subjects consented to their data being used. Nine failed the attention check. We removed three subjects because all of their weights on advice were either above two or below negative two, following prior literature and our preregistration (3). We removed 67 subjects because they exclusively put either no weight on advice or exactly the advice for every question. Multiple subjects emailed us explaining that they thought the instructions, which said to “note the source of the advice”, meant that they were supposed to simply write the advice itself rather than their best estimate. We did not use a manipulation check for this experiment, because subjects were exposed to both sources of advice. We ran our models on the remaining 514 subjects.

In experiment three we used the same procedure for recruiting and excluding participants as we did in experiment one and two. We did not allow subjects who completed either experiment one or experiment two to participate in experiment three. We started with 673 responses. 100 of those respondents claimed they were in the army, and thus we did not allow them to complete the experiment (due to limitations imposed by our funding). Of those 573, four did not consent to our use of their data. 73 subjects failed the attention check. 39 subjects either exclusively took the advice

without consideration for their initial estimate (WOA always equal to one) or exclusively disregarded the advice completely (WOA always equal to 0). We ran our models on the remaining 461 subjects.

2.5.2 Analytical Approach

As with our analyses on weight on advice, we again used multilevel mixed-effects linear regression with random intercepts – fit using the lme4 package in the R computing environment (4) – to analyze the effects of the advice type and task difficulty on weight on advice, time, and confidence. For the models with the change in time spent as the dependent variables, we control for both the initial confidence in an estimate prior to seeing advice, and for accuracy prior to advice. For the model with the change in confidence as the dependent variable, we do not control for initial confidence so as to not include initial confidence on both sides of our structural equation. Our main model:

$$y_{ik} = \beta_{0i} + \beta_1 \text{AlgoCondition}_i + \beta_2 \text{Difficulty}_k$$
$$+\beta_3 \text{AlgoCondition}_i \times \text{ Difficulty}_k + \beta X_{ik} + \varepsilon_{ik}$$

Here, y_{ik} is one of the dependent variables for participant i and problem k; β_{0i} is the slope for participant i; AlgoCondition_i and Difficulty_k are dummy variables indicating the advice condition and problem difficulty respectively; and X_{ik} is a vector of control variables. As with the main analysis, we also included terms for advice quality and the corresponding interactions for experiment three.

2.5.3 Variables Unique to the Supplemental Analysis:

Time Change: The percentage change in the amount of time taken to submit the page, between the first answer and second answer. It includes both the time taken to answer the question about crowd size and time taken to submit confidence. A negative value indicates the subject spent less time on their answer after receiving advice than on their answer prior to receiving advice.

Confidence Change: the percentage change in confidence between and initial answer and an answer after advice was received. A negative value indicates a subject became less confident after receiving advice.

3 NORMALITY ANALYSIS

We assessed the appropriateness of using linear mixed-effects models on our Weight on Advice variable by plotting the theoretical quantiles of the random intercept against the sample quantiles (Figure S3a below) and the standardized residuals against the quantiles of the standard normal (Figure S3b below). These plots indicate that a linear mixed effects model is an appropriate tool to analyze our data.

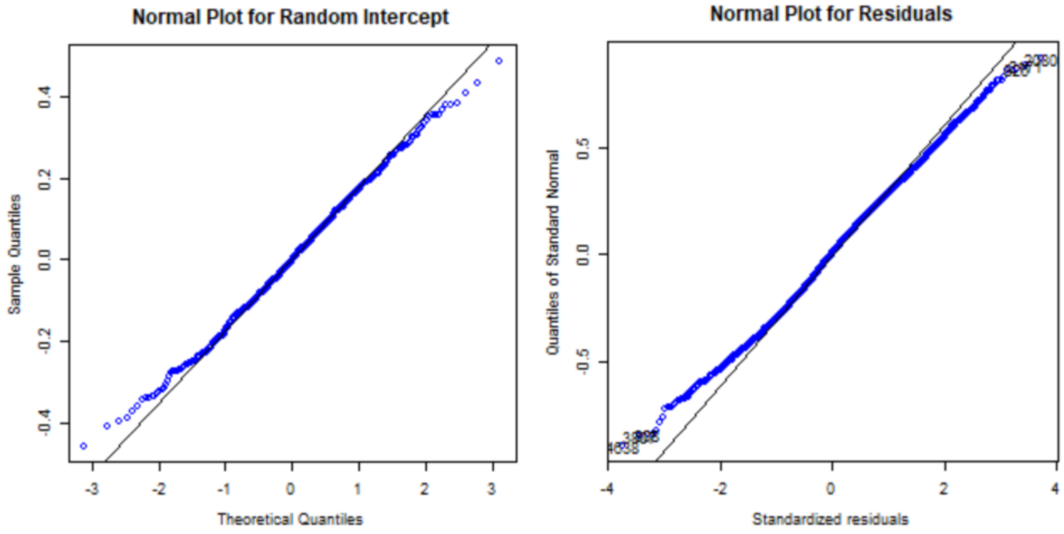

(A) (B)

Figure S3. Plotting theoretical quantiles against sample quantiles (A) and standardized residuals against the normal distribution (B)

4 CONSENT AND DEBRIEF MATERIALS

4.1 Consent Form

Dear Participant,
My name is Aaron Schecter and I am a faculty member in the Management Information Systems Department at the University of Georgia. I am inviting you to take part in a research study. I am doing research on how individuals work in teams to complete problem-solving tasks. Specifically, I am investigating how different technologies such as artificial intelligence or virtual communication can affect teamwork. Your responses may help us understand how teams can function effectively while using these advanced technologies.
I am looking for individuals age 18 and older who are residents of the United States and not current military personnel.
If you agree to take part in this study, you will be asked to view an image and assess how many elements are contained in that image. As part of the decision-making process, you will be shown an image twice. The first time you see it you will not have any advice. Your guesses may be visible to future participants. In order to make this study a valid one, some information about the study will be withheld until completion of the task. You will also be asked to complete a set of survey questions regarding your experience. This task should take approximately ten minutes, including time to answer survey questions. Participation is voluntary. You can refuse to take part or stop at any time without penalty. Your decision to participate will have no impact in your participation in future studies. You may find the task difficult or frustrating. There is no penalty for submitting an incorrect answer or not completing the task accurately. If any survey questions make you uncomfortable, you can skip these questions if you do not wish to answer them.
Research records will be labeled with study IDs only. There will be no record of your name or other identifiable information. Because all responses are anonymous, the information may be used in future research studies or shared with other researchers without additional consent. This research involves the transmission of data over the Internet. Every reasonable effort has been taken to ensure the effective use of available technology; however, confidentiality during online communication cannot be guaranteed.
As compensation for your participation, you will receive money in your worker account. For completing the task, you will receive $1.50. **We will give more money, in the form of a bonus, to workers who are more accurate.**
This research is supported by the Army Research Office. Representatives of the Department of Defense are authorized to review research records.
If you are interested in participating or have questions about this research, please feel free to contact me at aschecter@uga.edu. If you have any complaints or questions about your rights as a research volunteer, contact the IRB at 706-542-3199 or by email at IRB@uga.edu.
Please keep this letter for your records.

Sincerely,
Aaron Schecter

4.2 Debrief Form

Thank you for your participation in this research study. For this study, it was important that we withhold information about some aspects of the study. Now that your participation is completed, we will describe the withheld information to you, why it was important, answer any of your questions, and provide you with the opportunity to make a decision on whether you would like to have your data included in this study.

What you should know about this study
During the study, you were able to view the guesses of other participants or the guess of an algorithm and factor them into your decisions. In reality, the only difference was the label, the advice was always the same. All participants, including yourself, viewed the same images, and no participant had an advantage over another. Not explaining that the advice was identical is an important component of this study. Humans typically interact with other people differently than they interact with technology, and they have different expectations about what each are capable of. By keeping the advice identical but labelling it differently, we can determine if your actions and perceptions are dependent on the label “algorithm". This research helps us better understand how beliefs regarding AI technology affects problem solving and teamwork.

Right to withdraw data
You may choose to withdraw the data you provided prior to debriefing, without penalty or loss of benefits to which you are otherwise entitled. Please check the box below if you do, or do not, give permission to have your data included in the study:

_______I give permission for the data collected from or about me to be included in the study.

_______I DO NOT give permission for the data collected from or about me to be included in the study.

Whether you agree or do not agree to have your data used for this study, you will still receive the $1.50 in your worker account.

Disclosure
Please do not disclose research procedures and/or purpose to anyone who might participate in this study in the future as this could affect the results of the study. This includes online forums, message boards, or social media sites.

If you have questions
The main researcher conducting this study is Aaron Schecter. If you have questions, you may contact the IRB at 706-542-3199. If you have any questions or concerns regarding your rights as a research participant in this study, you may contact the Institutional Review Board (IRB) Chairperson at irb@uga.edu.

5 SUPPLEMENTARY REFERENCES

1. V. Alexander, C. Blinder, P. Zak, Why trust an algorithm? Performance, cognition and neurophysiology. *Comput. Human Behav.* **89**, 279–288 (2018).
2. J. Simmons, L. Nelson, U. Simonsohn, False-Positive Psychology: Undisclosed Flexibility in Data Collection and Analysis Allows Presenting Anything as Significant. *Psychol. Sci.* **22**, 1359–1366 (2011).
3. J. Logg, J. Minson, D. Moore, Algorithmic Appreciation: People prefer algorithmic to human judgment. *Organ. Behav. Hum. Decis. Process.* **151**, 90–103 (2019).
4. D. Bates, M. Mächler, B. Bolker, S. Walker, Fitting Linear Mixed-Effects Models Using lme4. *arXiv Prepr.* (2014).
